# Influence of Synthetic Bone Substitutes on the Anchorage Behavior of Open-Porous Acetabular Cup

**DOI:** 10.3390/ma12071052

**Published:** 2019-03-30

**Authors:** Volker Weißmann, Tim Ramskogler, Christian Schulze, Rainer Bader, Harald Hansmann

**Affiliations:** 1 Faculty of Engineering, University of Applied Sciences, Technology, Business and Design, Philipp-Müller-Str. 14, 23966 Wismar, Germany; h.hansmann@ipt-wismar.de; 2 Biomechanics and Implant Technology Research Laboratory, Department of Orthopedics, Rostock University Medicial Center, Doberaner Strasse 142, 18057 Rostock, Germany; christian_schulze@med.uni-rostock.de (C.S.); rainer.bader@med.uni-rostock.de (R.B.); 3 Department Industrial Engineering, Technical University of Applied Sciences, Hetzenrichter Weg 15, 92637 Weiden, Germany; tim.ramskogler@web.de

**Keywords:** Ti6Al4V, selective laser melting, press-fit, primary stability, bone substitute material

## Abstract

Background: The development in implants such as acetabular cups using additive manufacturing techniques is playing an increasingly important role in the healthcare industry. Method: This study compared the primary stability of four selectively laser-melted press-fit cups (Ti6Al4V) with open-porous, load-bearing structural elements on the surface. The aim was to assess whether the material of the artificial bone stock affects the primary stability of the acetabular cup. The surface structures consist of repeated open-porous, load-bearing elements orthogonal to the acetabular surface. Experimental pull-out and lever-out tests were performed on exact-fit and press-fit cups to evaluate the primary stability of the cups in different synthetic bone substitutes. The acetabular components were placed in three different commercially available synthetic materials (ROHACELL-IGF 110, SikaBlock M330, Sawbones Solid Rigid). Results & conclusions: Within the scope of the study, it was possible to show the differences in fixation strength between the tested acetabular cups depending on their design, the structural elements used, and the different bone substitute material. In addition, functional correlations could be found which provide a qualitative reference to the material density of the bone stock and the press-fit volume of the acetabular cups.

## 1. Introduction

The development of new implant designs using additive manufacturing technologies [1,2,3,4,5,6] and the integration of property-improving design elements, such as open-porous structures for the treatment of large and small segmental bone defects [7,8,9], requires, inter alia, a verification of suitability for the declared objective. The functional properties of the implants must be demonstrated in addition to surface quality [10,11], cell biological compatibility [12,13], corrosion resistance, and mechanical strength [14,15], which are significantly influenced by the material used. These include the primary fixation strength, when the implant is placed in the bone, where it plays a decisive role in determining the success of an implantation, i.e., an insertion of an artificial acetabular cup into the bone stock [16].

The application of open-porous, additive structures to human biomedical problems [17] is now widespread and ranges from dental [18] to orthopedic [19] applications. In addition to selective laser melting (SLM process), electron beam melting (EBM process), is a widely used process with confirmed suitability for the production of components for applications having a biomedical background [20,21].

Dental applications here focus on the replacement of individual teeth, the jaw or entire jaw segments [22,23]. Orthopedic applications in the field of hip and knee endoprostheses [24,25,26,27,28] already offer a number of examples that aim to fulfil load-bearing functions. In the area of artificial acetabular cups, solutions exist that achieve mechanical properties in the range of cancellous bone [29,30] or offer structures that especially support bone ingrowth [31] and thus primarily improve secondary stiffness. 

Cementless press-fit designs are in direct competition with cemented solutions. However, no significant influences of the fixation method could be demonstrated in a metastudy conducted by Toossi et al. on total hip replacement or in a study by Bjørgul et al. on a cemented and an uncemented hip cup [32,33]. In addition to applications of geometrically defined scaffolds on implants, efforts are being made to develop metallic interconnecting cell systems [34] or meshes [35] for biomedical applications. 

Of particular interest in preclinical studies is the fixation strength of implants. Therefore, experimental work studying the detachment of implants from the surrounding bone or bone substitute material are common.

In the field of orthopedic implants, it is mainly pull-out and lever-out tests that are used to describe the primary fixation strength [36,37,38,39]. Methods that, for example, allow the evaluation of friction forces [40] are, however, also suitable for a quantitative description.

In addition to investigations using bone (cadavers or corpse donation) [41,42], closed-cell rigid plastic foams are increasingly being used to assess fixation strength [36,43]. Although closed-cell rigid foams deviate from the properties of acetabular bone, they are very convenient for the evaluation of primary fixation strength due to the mechanical properties resulting from the uniform cell structure. In addition, these foams are more readily available. The use of these foams also avoids ethical problems and allows reproducible delivery of the foams. With the artificial bone support materials, different qualities as well as osteoporotic and sclerotic bone can be simulated [44,45,46].

The successful osseointegration of an implant is fundamentally decisive for the success of implant integration into the human organism. Successful implantation and good long-term results can only be expected if the transition from primary to secondary stability of the implant in the surrounding bone merges seamlessly [47,48]. A sufficiently high postoperative stability, i.e., primary stability, must be ensured as a basic prerequisite for permanent and sufficiently firm ingrowth [49]. Primary stability is influenced by the bone properties (density, structure) of the implant site as well as by the implant itself. The implant can be designed with reference to the existing conditions in a variety of ways by the material used, the surface structure and coating, as well as the shape.

Investigations to evaluate the secondary strength are mainly carried out in animal experiments [50,51,52]. Here, in addition to the evaluation of the load-bearing function, are excellent possibilities to qualitatively and quantitatively evaluate bone ingrowth depending on the implant design and its geometric conditions as well as possible functional surface modifications [53].

The constructional transfer of open-porous, functionally designed structural elements to implants that assume load-bearing functions in the bone thus offers enormous potential but also confronts the developer with equally enormous challenges.

The aim of this study is to evaluate the fixation strength of additively manufactured press-fit acetabular cups in artificial bone cavity. The basic aim is to improve the understanding of the artificial bone materials used and thus to possibly create a better starting situation for implant development in preclinical studies. Press-fit acetabular cups were designed accordingly with open-porous and differently dimensioned structural elements and characterized experimentally in artificial bone cavities with varying material properties.

## 2. Materials and Methods

### 2.1. Cup Design

**Figure 1:**
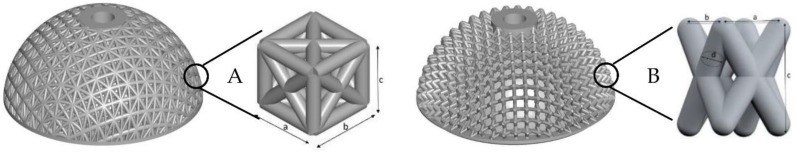
Illustration of the artificial press-fit cups, which are constructed with an open-porous, load-bearing unit cell; (**A**)—combined unit cell, (**B**)—twisted unit cell.

For the investigations, four modified press-fit cups with flattened poles (Figure 1) were designed and manufactured. The structural elements and bodies as well as the implant models were constructed using the software PTC Creo, Version 3.0 (Parametric Technology Corporation, Needham MA, USA). The cup variants V (twisted unit cell geometry) and D (combined unit cell geometry) are identical in dimensions a and b. A variation was made in the diameter of the individual rods in variant V (0.9 and 1.0 mm) as well as in variant D (0.8 and 0.9 mm). The twisted structure consists of interlaced rod elements in the form of an X. The combined unit cell geometry consists of a cubic structure with transverse struts on the outer surfaces and an inner structure. The inner structure is diamond-like.

Very important to characterizing the function of components with open-pore elements are parameters that influence the mechanical properties, the anchorage behavior or the ingrowth behavior. In addition to porosity, this also includes the volume causing the press-fit in the artificial bone cavity [20,26,54]. 

The porosity of the load-bearing and structure-determining layer was determined directly from the CAD data as the quotient of the structure volume and the full volume. The volume that generates the press-fit after insertion of the acetabular cup into the artificial bone cavity was also determined from the CAD data. The size of the volume inside the artificial bone cavity was quantified virtually. A detailed description of the calculations can be found in Weißmann et al. [54]. The results for every cup design are listed in Table 1.

### 2.2. Fabrication

(1) All cups were manufactured by C.F.K. CNC-Fertigungstechnik Kriftel GmbH (Kriftel, Germany). The SLM process was used in a highly pure argon atmosphere. An SLM 280 system and titanium powder (Ti6Al4V) with a mean particle size of 43.5 µm, which meets the requirements of ASTM F 67, were used. All cups were manufactured using identical manufacturing parameters and orientation in the implantation area. The CAD data are the basis for manufacturing. The parameters, the pre- and post-treatment of the parts correspond to the description in [54].

(2) Two polyurethane foams (Sawbones pcf 20 and SikaBlock M330) and one polymethacrylimide rigid foam (ROHACELL-IGF 110) were used as synthetic bone substrate.

The properties of SikaBlock M 330 (Sika GmbH, Bad Urach, Germany), Sawbones pcf 20 (Sawbones Europe AB, Malmö, Sweden) and ROHACELL-IGF 110 (Evonik Röhm GmbH, Darmstadt, Germany) are shown in Table 2. The material properties were determined using applicable standards. The compressive strength was determined using the ISO 844 test standard [55], the density was determined using the ISO 845 test standard [56], and the modulus of elasticity was determined using the ISO 844 test standard [55].

The material (SikaBlock and ROHACELL) was supplied in plates of 1000 mm × 500 mm. The dimensions of the Sawbones material were 180 mm × 130 mm × 40 mm. Blocks were cut out of the foam plates with side lengths of 100 mm × 100 mm × 50 mm (SikaBlock and ROHACELL) all along the panel width so as to cover possible fluctuations of the compressive modulus in this range. The Sawbone materials were halved for the production of the cavities. 

The artificial bone cavities were made using the material blocks. A CNC milling machine i-mes-FLATCOM 50-VH (i-mes GmbH, Eiterfeld, Germany) was used. The modulus of elasticity (SikaBlock and ROHACELL) increases from the center of the block to the outer edge due to production. To take this influence into account, the experimental design was adapted and the bone cavities were used in rows. Each cup variant was tested along the entire material line (n = 5). Any differences in the modulus of elasticity were taken into account as described in Weißmann et al. [62].

### 2.3. Measurements

The measurements conducted are briefly explained below. A more detailed description can be found in the publication by Weißmann et al. [54].

**Figure 2:**
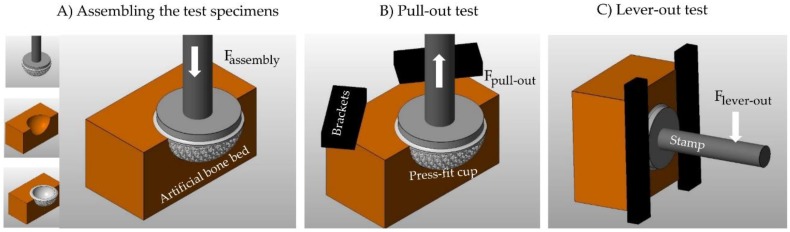
Schematic representation of the experimental test environment for determining the fixation strength. (**A**) Inserting the press-fit cup into the artificial bone cavity; (**B**) Pull-out test; (**C**) Lever-out test.

(1) The acetabular cups and artificial bone cavities were measured using a noncontact measuring microscope (Mitutoyo-QVE-200 Pro; Mitutoyo Corporation, Kawasaki, Japan). The measured values were in each case circles and the best possible fit was determined using the least squares method. Possible measurement data outliers, which could be caused by light reflections and loose PUR particles, were removed using a box plot (according to John W. Tukey) in a Matlab script. The measurement results (resulting replacement diameter) were then used to determine the press-fits.

(2) The characterization of the primary stability (fixation strength) of the press-fit cups (Figure 2) was based on pull-out tests (2.1) and lever tests (2.2).

The acetabular components were inserted into the prepared synthetic bone blocks using a custom-made mount for the cups, which can be clamped into the machines clamping system to enable optimal conditions for the seating process. A uniaxial material testing machine INSTRON E 10,000 (Instron GmbH, Darmstadt, Germany) assembled with a 10 kN load cell and a Zwick Z50 (Zwick GmbH & Co. KG, Ulm, Germany) assembled with a 50 kN load cell were used to seat the acetabular cups at a linear rate of 5 mm/min. The cups considered to be seated when the top rim of the acetabular component fit flush with the top surface of the foam block. This position was then checked with a 0.1 mm feeler gauge pushed against the equatorial rim of the acetabular cup during the seating process until it slides over the top rim. Only in this position can the optimum size of press-fit be obtained.

(2.1) After the seating process, the cavity including the seated acetabular component was fixed with a special clamping device. The uniaxial testing machine (INSTRON E 10,000) was used to directly dislodge the acetabular component from the cavity at a rate of 5 mm/min.

(2.2) Following the seating process the custom-made mount with the acetabular cup and the currently fixed cavity was disconnected from the load cell of the Zwick/Roell Z050. It was reconnected to a clamping device consisting of different metal blocks that were attached in a way that avoided the fixed cavity block including the still attached acetabular cup and custom-made mount from tilting forward or sideward. A pressure attachment was fixed to the load cell. The effective moment arm for this test was defined as the distance from the top surface of the cavity, and therefore the top rim of the acetabular cup, to the point of contact of the attachment with the mount. The pressure attachment was lowered at a rate of 5 mm/min to tilt the acetabular cup out of the fixed cavity. 

(3) A digital microscope—Keyence VHX 2000 (Osaka, Japan)—was used for the optical evaluation of the artificial bone cavities. Following the milling process, first a qualitative evaluation of the surface was carried out. Then the surface was recorded 3-dimensionally and displayed in false colors. This display enables the quantitative (based on the height marks) and qualitative evaluation of the traces left in the bone cavity by the press-fit cup with the porous structures. 

**Figure 3:**
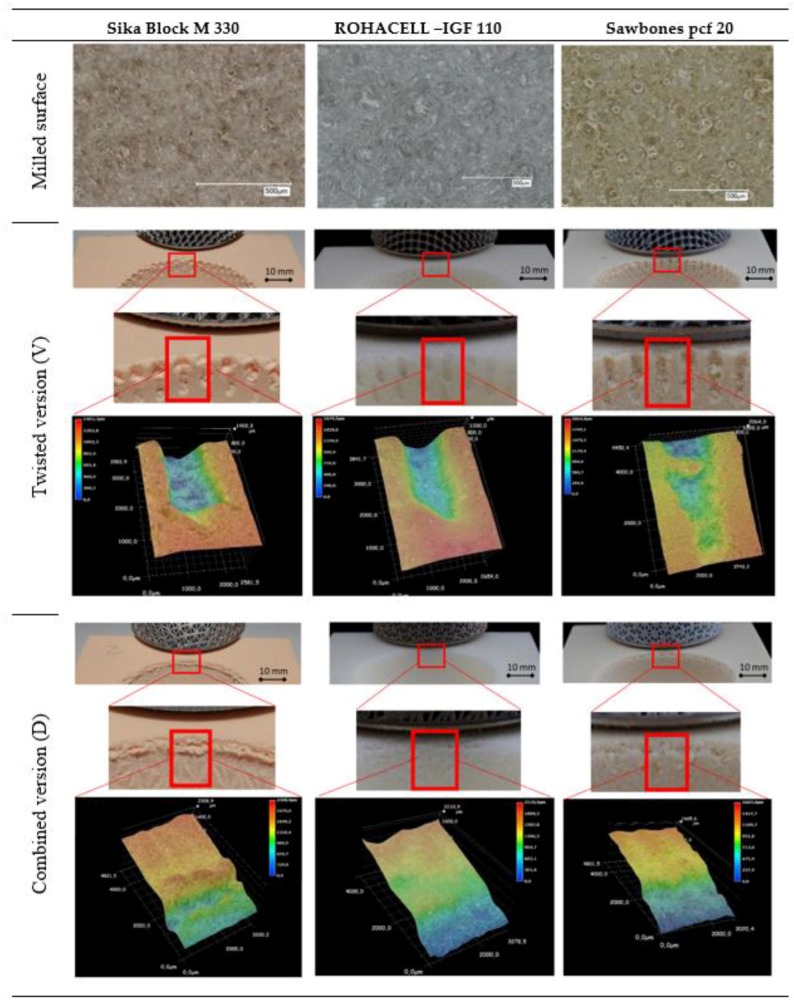
Representative images of the various artificial bone cavity materials after cup seat creation using machine milling and of the twisted structure and the combined structure after the testing to determine fixation strength.

These demonstrations were made using representative examples (Figure 3) for the pull-out and lever-out test on the basic structures used (twisted and combined). 

### 2.4. Statistical Analysis

All data listed in the tables are expressed as mean values ± standard deviation (SD). The relationships between the ratio of the press-in force and the pull-out force or lever-out force to the volume of the press-fit area as well as the ratio of the press-in force and the pull-out force or lever-out force to the density of the artificial bone cavity materials were evaluated by nonlinear regression with Excel 2016 for Windows. All statistical analyses were performed with GraphPad Prism 7.02 for Windows (GraphPad Software, La Jolla, CA, USA). The pull-out force and lever-out forces were also evaluated statistically. In order to determine significant differences, the means were evaluated using a one-way ANOVA with a Bonferroni’s post hoc test. The *p*-values determined for the paired comparisons were presented in a table. *p* < 0.05 was considered statistically significant.

## 3. Results

### 3.1. Accuracy of Fabricated Samples

Table 3 lists the dimensions measured for the acetabular cup as well as the press-fit values determined for the possible pairings of bone cavity and cup. 

The press-fit results were determined using the measurement results for the artificial bone cavities and the dimensions of the press-fit cups. In all cases, a press-fit of 2 mm was constructively aimed for. The dimension for the press-fit was chosen on the basis of commercially available cups and based on the result of preliminary tests. In the preliminary tests, the best results for fixation strength were achieved with a 2 mm press-fit.

The measurement results for the press-fits determined vary between 2.02 mm and 2.09 mm. The deviations for the Sawbones and ROHACELL materials between the different cups is not greater than 0.01 mm. The SikaBlock materials show the largest deviation between each other with 0.07 mm. The deviation from the smallest possible press-fit here is less than 4% (3.46%). The bone cavities deviate only slightly from ideal circles (roundness values 0.11 to 0.30). This is clear evidence of the accuracy of the production. With respect to the additive manufacturing process, these are excellent results [63,64,65,66].

### 3.2. Initial Stability

In order to evaluate initial stability, pull-out and lever-out tests were conducted. For this purpose, the cups were removed after insertion into the bone supports. The seating forces and the results for the pull-out forces and the lever-out forces are shown in Table 4. The statistical results are listed in Table 5.

The seating process was concluded without any issues. The seating forces in ROHACELL were in a range between 1813 N to 2669 N, in SikaBlock between 1952 N to 4339 N and in Sawbones between 4551 N to 6528 N.

The results show that there are differences due to the structural elements and the artificial bone bearing materials used. In both fixation strength tests and in the three artificial bone cavity materials used, the cups with the combined structures achieved the best results. The greatest forces when pulling and tilting the cups out of the bone cavities are required in the Sawbones material, followed by SikaBlock and ROHACELL. 

It is noticeable that the differences between the cup types in SikaBlock and ROHACELL material are rather small. Only the Sawbones material shows clear differences in the measurement results. This applies to the cups with the twisted structure as well as to those with the combined structure.

Using the results of the statistical significance test conducted (one-way ANOVA with Bonferroni’s post hoc test-multiple comparisons), the following statements can be formulated. 

The cups with the twisted structures show no significant differences in comparison to each other for all materials used, and in both experiments. The cups with the combined structures show only significant differences when compared in between the Sawbones material. When comparing the twisted and combined cup structures, the results are somewhat more differentiated. In the Sawbones and SikaBlock material, the cups with the twisted structures achieve significantly lower values in the pull-out and lever-out tests than the cups with the combined structures. In the Rohacell material this relationship is only valid for the pull-out tests. In the lever-out test only the cup variant V4_10 deviates significantly from the cups D4_08 and D4_09.

### 3.3. Microscopy

To evaluate the quality of the artificial bone cavity materials used, micrographs were obtained for the various press-fit acetabular cups. Images were first taken of the materials used after machining. Then, images representative for the traces left by a twisted structure and a combined structure were taken after the tests to evaluate the fixation strength in the artificial bone cavity. The 3-dimensional images for this purpose were displayed digitally in false colors. In addition, a section of the bone cavity carrying the respective traces of the acetabular cups was shown. These images are seen in Figure 3. The presentation of possible differences between the pull-out and lever-out tests has been omitted here, as the current aim was to show the basic material behavior as a function of the cup structure. The images were taken at the upper edge of the artificial bone cavity. This is where the press-fit is the most effective.

While the SikaBlock material structures appear rather blurred and only isolated traces of a foam structure can be seen, the Sawbones material clearly shows the structure of a foam. After the cavities required for the acetabular cups have been created by milling, clear structures are identifiable and individual, open spherical foam particles are visible. This material shows a clear and uniformly machined surface. The ROHACELL material also shows clear individual elements of the foam. However, here too, an uneven surface structure similar to the SikaBlock material can be seen. The foam particles themselves can be identified here more as polygonal elements.

Looking at the traces left by the individual acetabular structure elements after their detachment from the artificial bone cavites, clear differences can be seen in the false color representations. The twisted structure produces a rather clearly defined trace of the element in the Rohacell material. Here, the material appears rather compressed. Destructions on the surface are not visible. This is also reflected in the rather even color over the entire surface. The Sawbones and SikaBlock materials clearly behave differently here. Especially in the Sawbones material, jumps in the elevation profile are recognizable, indicating that the material behaves distinctly differently locally. On one hand, it becomes clear that locally the material can adapt to the structure and, due to its own stiffness, on the other hand, can react very differently to the mechanical loads (resulting from the press-fit process and subsequent test). This difference is also visible for the SikaBlock material, but in an attenuated form. These descriptions apply equally to the pull-out and lever-out tests. In this context the combined structure leaves a coherent structural trace which nevertheless indicates the defined structure of the unit cell clearly.

### 3.4. Correlations—Lever-out Moment and Pull-out Force versus Density and Volume of the Press-Fit Area

**Figure 4:**
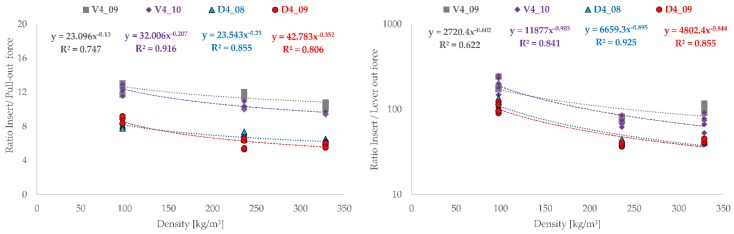
Representation of press-in ratio to the forces determined experimentally for the pull-out test and lever-out test in comparison to the material density (SikaBlock = 236 kg/m^3^, Sawbones = 328.6 kg/m^3^ and ROHACELL = 97.6 kg/m^3^) of the artificial bone cavities (n = 5).

**Figure 5:**
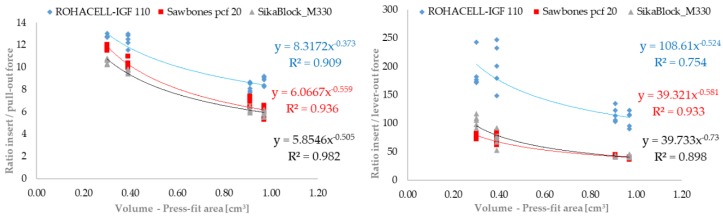
Representation of press-in ratio and to the forces determined experimentally for pull-out test and lever-out test in comparison to the press-fit volume of the tested acetabular cup variant (n = 5).

The ratios of the forces required to press the cups into the artificial bone cavities to the pull-out and experimentally determined lever-out forces are plotted graphically over the density of the bone cavity materials Figure 4 and over the volumes Figure 5 responsible for the press-fit. 

Basically, the tested cup types show identical behavior with regard to the results. The order of the magnitude of the ratios (pull-out—8 to 10; lever-out approximately 100) differs between the test procedures, but in both cases shows that the twisted structures and the combined structures each exhibit the same result level. The tested cups can be described very well functionally by power law and show good regression coefficients. The cup with the twisted structure V4_09 the smallest similarities with a R^2^ = 0.747 for the pull-out tests and R^2^ = 0.622 for the lever-out tests. On one hand, it becomes clear from the progressions that a high density of the bone cavity material correlates with low ratios between press-in force and pull-out or lever-out force. With reference to the experimental results, it also becomes clear that low ratios at high densities are synonymous with good fixation strength.

The graphic evaluation of the pull-out and lever-out behavior in the form of the determined conditions (insertion and removal from the artificial bone cavity) plotted over the relevant press-fit volume shows uniform curves. The functional behavior of the tested acetabular cups can be described excellently using the power law. For both test forms (pull-out and lever-out), the results of the cups for the ROHACELL material differ slightly from those of the other materials. This difference is more clearly visible in the lever-out test than in the pull-out test. The results lie very close for the SikaBlock and Sawbones material in both tests. The regression coefficients determined are slightly higher in the pull-out test (0.909; 0.936 and 0.982) than in the lever-out test (0.754; 0.933 and 0.898). In both cases, however, they prove the possibility of describing the anchorage behavior with the assistance of the volume responsible for the press-fit.

## 4. Discussions

The motivation for this study is to evaluate of the anchorage behavior of differently dimensioned and designed press-fit acetabular cups in artificial bone materials. The manufacturing accuracy of the cavity and cup already plays an important role here.

The forces required to press in the cups are also influenced by dimensional differences in the press-fit. This may result in uneven conditions for contact between the press-fit cup and the surface of the bone cavity and stress differences in the bone cavity. These differences lead to different conditions for the movement behavior of the press-fit cup in the bone bearing [41,46]. Good long-term results are only possible if the conditions for achieving good primary stability are fulfilled [67]. The press-fit chosen in this study is the result of an internal preliminary study. It is further known that other studies have told good results with similar values [68,69]. 

In this study, it can be assumed that the differences in press-fit between each other (SikaBlock 2.02 to 2.08; Sawbones 2.02 to 2.03; ROHACELL 2.02 to 2.03) are so small that this has no influence on the assessment of the primary stability of the artificial acetabulum. 

It is known that in the osseointegration phase, the stability of the acetabular components directly depends on the initial stability. A seamless transition from initial to secondary stability leads to long-term stability [59,70,71,72]. Studies have shown, that the availability of cancellous bone material, a large surface, a strong bone/implant interface and minimal motion of the implant directly after insertion are criteria for the optimal biological fixation of implants [73]. Therefore, the primary stability is the decisive property which can be tested in advance during in-vitro experiments. To acquire a strong bone–implant interface, a correctly conducted seating procedure is particularly important. An insufficient fit of the acetabular component in the cavity would lead to remaining pressure forces that cause excessive micromotions and therefore affect the primary stability [59,71,74]. 

In the literature, several methods of seating are used. One of these methods is a visual monitoring of the seating process. The components are considered to be seated, when the whole area of the component is covered by the bone material, such that only the top and the inner sleeve of it is visible [59]. The seating process in this study has been optimized by a haptic aspect. A 0.1 mm feeler gauge is used to control the optimal fit in the cavity. This position is reached, when the gauge slides over the top rim of the acetabular component. Prior studies have mostly compared the initial stability of acetabular components differing in geometry, surface design, press-fit size or other factors [36,48,59,60]. These studies show that the different acetabular components have different values for seating forces or primary stability. In contrast, this study investigates differences between the chosen artificial bone materials. Consistent with the hypothesis, it was observed that the material of the artificial bone leads to different seating forces and initial stability. Providing that seating have been conducted properly different seating forces has been recorded between the five acetabular components, but also for the three artificial bone cavities. 

It should also be noticed that the seating procedure of this current study is a manageable method to provide similar test conditions for every single cavity. An accurate monitoring of seating forces and primary stability is enabled. This is also the reason why the cavities are milled by a milling machine. In this way, every cavity has a perfect size in the tolerance range of 0.1 mm and an optimal condition for the press-fit can be provided. The equatorial area especially is an important sector of the press-fit which is also shown by the abrasion of the foam material on the equatorial rim of the acetabular component after the experiments. Of particular importance, therefore, is a precise edge of the cavity in the foam. 

The traces that the cups leave in the material during the experimental process correlate with the parameters of the experimental results. The integration of an optical evaluation of the artificial bone cavity is well known [11,62]. Cups with good fixation strength results (i.e., high pull-out forces) also show clearer traces in the bone cavity. This applies to the different material types as well as to the characteristics of the acetabular cups. In particular, the traces visualization in the cavity in false colors provides an excellent impression of the behavior of the structural elements in the cavity.

The destroyed and partially compressed areas caused by the individual elements of the twisted structural elements in the SikaBlock or Sawbones materials are clearly visible. The local differences here give a rather irregular appearance, but also give proof of the good adaptation of the elements to the bone cavity and the remaining structure (the material does not loosen). ROHACELL, on the other hand, displays a rather exclusively compressed image which correlates with the low forces that were determined in the pull-out and lever-out tests. These observations apply to both structural geometries (twisted and combined). Since the images were taken in the area of the bone cavities, it must be taken into account that the spherical progressions are naturally part of the images. This is particularly visible in the images for the combined structure (inclined image).

As well as the seating forces, the primary stability of the acetabular components obviously increases with denser material. The gathered values of the pull-out forces in SikaBlock are more than twice that of the values in ROHACELL. The values for Sawbones are 37% higher than the values for SikaBlock. Also, the values of the lever-out moments show an increase of 175% from ROHACELL to SikaBlock. The values for Sawbones are more than twice those for the values of SikaBlock. Consistent with the hypothesis, the recorded values show big differences between the different bone materials. The level of pull-out forces in this study is comparable to the range reported in previous studies (150 N to 1600 N) [75,76].

The results are substantiated by the statistical investigation. For the Sawbones material, clear differences between the different cup structures and between the tests become visible. While for the use of the Sawbones material, significant differences are shown for the cup variants D4_08 and D4_09, differences cannot be shown between the cup V4_09 and the combined variants (D4_08; D4_09) for the ROHACELL material. The Sawbones material therefore provides excellent results when differences are to be made visible.

Also clear is that with the Sawbones material, it is possible to determine high differences (e.g., pull-out force V4_09 = 386 N; V4_10 = 508 N) in the forces and thus to make differences in the cup design perfectly visible. Conversely, the ROHACELL material provides only small differences in results (e.g., pull-out force V4_09 = 147 N; V4_10 = 157 N) and therefore does not allow a differentiated view of the constructively generated differences in the selected test arrangements. With the SikaBlock material it is possible to display differences between the fundamental design variations (e.g., pull-out force V4_09 = 310 N; D4_09 = 704 N). The possibility of displaying geometric details in the experimental results is only very weakly possible here (e.g., pull-out force V4_09 = 310 N; V4_10 = 308 N).

In connection with the press-fit volume relevant for the fixation strength, it becomes clear that a low ratio between the press-in force and the experimentally determined force (e.g., Sawbones; ratio insert force/pull-out force—D4_08 = 6.88; press-fit volume = 0.91 cm^3^; V4_09 = 11.79 and 0.30 cm^3^) is a benefit. What also becomes clear is that a higher density (e.g., lever-out force/Sawbones—D4_08 = 168 N; SikaBlock—D4_08 = 83 N) has a positive effect on the assessment of fixation strength. Especially in connection with the development of implant structures, a high density seems helpful for the classification of different elements. Nevertheless, artificial bone materials with a lower density provide information that may be useful in connection with the development of implants and their structural elements. For instance, when poor anchorage conditions are to be simulated.

The functional description of the experimental results based on the press-in behavior and pull-out or lever-out tests as well as the known press-fit volumes or material densities represents an excellent tool for a faster and more comfortable development of implant structures. In addition, it becomes clear that a low ratio between the press-in force required and the forces achieved when releasing the cup from the bone cavity can be tantamount to a high fixation strength. Depending on the required press-in force, acetabular cups that require the lowest possible forces should be adopted.

The experimental in vitro investigations demonstrated a fundamental suitability of press-fit acetabular cups with load-bearing structures for achieving a high primary fixation strength. 

It would be interesting to see how the results turn out in comparison to standard hip implants without open-porous structures, since the primary fixation strength can be controlled by the choice of the structural element (open-porous structure), but also by the resulting press-fit volume. The production of acetabular cups with porous structures opens up the possibility of providing bone cells with optimal conditions for proliferation and nutrient transport [2,77,78]. For meaningful results, however, animal experiments [50,79,80] would be recommended, e.g., to also assess bone ingrowth behavior.

## 5. Conclusions

Experiments investigating the primary stability of acetabular cups in artificial bone are not comparable to intraoperative situations. Nevertheless, it can be expected that an acetabular component having high primary stability in laboratory investigations also performs better in human bone than a component having low primary stability. The artificial bone materials evaluated in this study offer a broad spectrum of densities and thus allow for the simulation of different bone qualities. In this study, press-fit acetabular cups with a porous supporting structural layer were investigated for primary stability.

The results show significantly different pull-out forces and lever-out forces in relation to the acetabular cups and artificial bone support materials, as determined experimentally. 

The press-fit cups D4_08 and D4_09 (combined structure) achieve the best results in pull-out and lever-out behavior. Herein, the Sawbones material stands out with excellent results as compared to the SikaBlock and ROHACELL materials. 

Based on the material densities and the volume responsible for the press-fit, the determined functional relationships offer the possibility of specifically influencing the development of press-fit acetabular cups. By using different bone substitute materials, acetabular components with lower (osteoporotic bone) and higher (healthy human bone) density can be represented.

## Figures and Tables

**Table 1:** Listing of the various cup designs based on the unit cell variants of the two investigated designs (twisted and combined) as well as of the unit cell dimensions, porosity, and volume of the press-fit area. The values listed were taken from the CAD data.

Dimensions Unit Cell	Twisted (V)		Combined (D)	
**Parameter**	**V4_09**	**V4_10**	**D4_09**	**D4_08**
Width—a (mm)	2.83	2.83	4	4
Depth—b (mm)	2.83	2.83	4	4
Height—c (mm)	4	4	4	4
Strut diameter—d (mm)	0.9	1.0	0.9	0.8
Porosity of the structure area (%)	72.5	67.4	61.1	66.9
Volume—press-fit area (cm^3^)	0.30	0.39	0.97	0.91

**Table 2:** Overview of the experimentally determined mechanical properties of artificial bone cavity materials in comparison to literature data on human bone [36,57,58,59,60,61].

Property	ROHACELL IGF 110	Sawbones pcf 20	SikaBlock M330	Human Bone
Density (kg/m^3^)	97.6	328.6	236	50–1000
Compressive modulus (MPa)	137–168	238–276	133–183	10–2000
Compressive strength (MPa)	3.2	8.4	3.4	0.9–9

**Table 3:** List of relevant dimensions for the press-fit cups and press-fits (bone bearing–press-fit cup = press-fit). The arithmetic mean results from the number of samples n = 5.

Name	Press-Fit Cup		Press-Fit (mm)					
	Best Fit Circle (mm)	Roundness (mm)	SikaBlock		Sawbones		ROHACELL	
V4_09	54.90	0.29	2.05	±0.02	2.03	±0.02	2.02	±0.02
V4_10	55.03	0.02	2.09	±0.02	2.02	±0.02	2.02	±0.03
D4_08	54.98	0.30	2.08	±0.01	2.03	±0.03	2.03	±0.01
D4_09	55.04	0.11	2.02	±0.01	2.03	±0.02	2.02	±0.01

**Table 4:** Results for the insertion processes in preparation of the pull-out and lever-out tests as well as the forces determined in the tests during the pull-out and lever-out of the cups. The values output as arithmetic mean were determined from n = 5 values.

	SikaBlock		Sawbone		ROHACELL		SikaBlock		Sawbones		ROHACELL	
**Cup**	**Seating Force/Pull-out (N)**						**Seating Force/Lever-out (N)**					
V4_09	3266	±218	4551	±574	1849	±46	2814	±308	6025	±855	1813	±86
V4_10	2971	±151	5248	±184	1943	±46	1952	±76	5504	±1012	1965	±123
D4_08	4339	±264	5459	±572	2635	±21	3775	±210	5475	±50	2460	±59
D4_09	4173	±135	6528	±1819	2669	±32	3495	±208	6445	±653	2408	±91
**Cup**	**Pull-out Force (N)**						**Lever-out Force (N)**					
V4_09	310	±24	386	±43	147	±7	24	±7	81	±15	10	±1
V4_10	308	±11	508	±21	157	±7	28	±4	78	±22	10	±2
D4_08	708	±38	793	±44	323	±13	90	±6	128	±4	21	±2
D4_09	704	±32	1181	±145	305	±10	83	±7	168	±19	23	±2

**Table 5:** Tabular listing of the *p*-values (one-way ANOVA with Bonferroni’s post hoc test) determined for lever and pull-out forces for the various press-fit cups and artificial bone bearings. The values of *p* < 0.05 were defined as significant (n.s.: not significant).

	Pull-out Force					Lever-out Forcece			
Bone material	Cup	V4_10	D4_08	D4_09		Cup	V4_10	D4_08	D4_09
ROHACELL	V4_09	n.s.	<0.0001	0.0002		V4_09	n.s.	n.s.	n.s.
	V4_10	-	0.0002	<0.0001		V4_10	-	0.0300	0.0029
	D4_08	-	-	n.s.		D4_08	-	-	n.s.
Sawbone	V4_09	n.s.	0.0010	0.0016		V4_09	n.s.	0.0080	0.0003
	V4_10	-	0.0020	0.0058		V4_10	-	0.0592	0.0101
	D4_08	-	-	0.0420		D4_08	-	-	0.0456
SikaBlock	V4_09	n.s.	<0.0001	<0.0001		V4_09	n.s.	<0.0001	0.0002
	V4_10	-	<0.0001	<0.0001		V4_10	-	0.0006	0.0005
	D4_08	-	-	n.s.		D4_08	-	-	n.s.
